# Intraoperative optical coherence tomographic findings in patients undergoing subretinal gene therapy surgery

**DOI:** 10.1186/s40942-020-00216-1

**Published:** 2020-05-01

**Authors:** Huber M. Vasconcelos, Brandon J. Lujan, Mark E. Pennesi, Paul Yang, Andreas K. Lauer

**Affiliations:** 1 Casey Eye Institute, Oregon Health & Science University, 3375 SW Terwilliger Blvd, Portland, OR 97239 USA; 2 Department of Ophthalmology and Visual Sciences, Paulista School of Medicine, Federal University of São Paulo, São Paulo, Brazil

**Keywords:** Intraoperative optical coherence tomography, Imaging, Gene therapy, Subretinal surgery, Vitrectomy, Inherited retinal diseases

## Abstract

**Background:**

To analyze intraoperative OCT (iOCT) findings during subretinal gene therapy.

**Methods:**

A single-center, retrospective, observational, case series study of twenty one eyes submitted to subretinal gene therapy. Intrasurgical high definition videos were included for analyzes. Cases with absence of iOCT video or unsuccessful bleb creation were excluded. Sharp needle tip (SNT) or blunted needle tip (BNT) and their interaction with neurosensory retina were evaluated. Presence of subretinal air bubbles, visible opened retinotomy, and medication reflux were also correlated and analyzed.

**Results:**

Nineteen of twenty-one eyes were included. Of the two excluded eyes, subretinal bleb creation was unsuccessful in one and technical issues prevented OCT image acquisition in the other. Immediately before subretinal injection, needle indention/penetration of the neurosensory retina with temporary indentation of the RPE/choroid was evident in 16 (84%) of the 19 eyes. Complete RPE/choroid indentation was needed with BNT use compared to SNT (p = 0.0114). An open retinotomy was identified in 14 (74%) of 19 eyes at the conclusion of bleb injection and was more commonly associated with SNT (p = 0.0108).

**Conclusions:**

iOCT provides valuable real-time feedback of cross-sectional retinal anatomy during subretinal gene therapy surgeries. The type of needle tip and its use during the gene therapy procedure seems to influence in the bleb creation and presence of visible open retinotomy. Further studies of iOCT findings during gene therapy delivery procedures are likely to help refine the surgical technique.

## Introduction

Inherited retinal dystrophies (IRDs) are a group of degenerative diseases caused by genetic defects that lead to a variety of phenotypes that collectively affect a large number of individuals world-wide [1, 2]. While numerous human clinical trials have been conducted for IRDs in the last two decades, few successful treatments have been identified. Once exception is the recently approved subretinal gene augmentation therapy with voretigene neparvovec-rzyl for biallelic RPE65 mutations. This treatment has ushered a new era where treatment of previously incurable IRDs might be possible [3, 4]. Along with effective vector design, the safe and effective delivery of these agents into the subretinal space has become a critical component for the successful gene therapy [5–9].

Intraoperative optical coherence tomography (iOCT) integrates spectral-domain OCT (SD-OCT) within an operating microscope and is considered a significant technological advance in retinal surgery [10–15]. In early studies, iOCT was used to evaluate subretinal delivery of retinal pigment epithelial cells in animals [16]. Newer iOCT methods in porcine eyes have measured subretinal bleb volume, pointed air bubble shadowing and described other findings such as triamcinolone leakage [17].

Early studies in patients examined surgical techniques and utility of the iOCT for gene therapy by evaluating the ability to identify foveal detachment, the degree of retinal stretch, the presence of air bubbles, incidence of injection into the suprachoroidal space, patency of retinotomies, the impact of shadowing by intraocular instruments and others surgical techniques aspects [18–23]. With the growing number of subretinal gene therapy trials, additional iOCT findings are being discovered. We evaluated the utility of iOCT during subretinal injection across multiple gene therapy trials and report novel findings.

## Methods

This was a retrospective, observational, case series study that evaluated the microscope-integrated iOCT findings in patients undergoing subretinal gene therapy surgery at the Oregon Health & Science University-Casey Eye Institute (OHSU-CEI) in Portland Oregon. The study followed the tenets of Declaration of Helsinki and complied with the requirements of the Health Insurance Portability and Accountability Act. All patients signed a statement of informed consent before enrollment, and all procedures were reviewed and approved by appropriate institutional review boards and ethics committees.

The inclusion criteria consisted of all patients enrolled for subretinal gene therapy surgery at OHSU-CEI from July 2017 to July 2018 for which iOCT was available during the surgery. Exclusion criteria were: inability to use iOCT during the procedure, failure to deliver the gene therapy medication and/or inability to create a subretinal bleb.

All subretinal gene therapy cases involved pre-operative planning with SD-OCT, fundus autofluorescence and kinetic and static visual field analysis. The volume and location of medication delivery were determined by study specific study protocols. The same surgeon performed all surgeries (AKL). An assistant retinal surgeon with previous experience in using the iOCT system acquired all iOCT images (HMV).

The OPMI LUMERA^®^ 700 surgical microscope with the integrated OCT camera RESCAN™ 700 (Carl Zeiss Meditec AG, Jena, Germany) system was used in all included patients. Using the CONSTELLATION^®^ Vision System (ALCON, USA), 23-gauge pars plana vitrectomy with induction of posterior vitreous detachment was performed in all patients. An extendible 41-gauge subretinal injection needle (23 gauge/0.6 mm; 1270.EXT; Dutch Ophthalmic RC, USA), or non-extendible 23 gauge needle with 41-gauge Teflon tip (Dutch Ophthalmic RC, USA) or 38-gauge subretinal PolyTip^®^ cannula (25 gauge/38 gauge 3219 MedOne Sarasota Florida USA) were used. Sharp or blunted needle tip, one-step subretinal bleb or pre-bleb technique (saline used to create an initial separation between the neurosensory retina and the RPE before the medication delivery), and manual or pneumatic-pedal assisted delivery methods were used, depending on the requirements of the gene therapy surgical protocol and surgeon technique choice for each case. The surgical protocol were directly linked to different type of gene therapy clinical trials and genotype/phenotype correlation as well as intrasurgical findings could not be provided. Based on surgeon choice, a regular blunted needle tip (BNT) or a sharp needle tip (SNT) were used. The SNT was created by manually trimming the BNT with a Vannas scissor. Less than 1 mm of the BNT was manually trimmed to create the SNT. For the automatic delivery method, the infusion pressure was controlled via foot-pedal and the maximum infusion pressure was 16 psi.

During the procedures, the operating surgeon worked in collaboration with an assisting retinal surgeon who navigated and acquired images from the treatment area using the iOCT. High-definition horizontal and vertical cross-sectional B-scans were recorded using real-time iOCT imaging during surgery. The iOCT videos were de-identified and reviewed by the surgeon (AKL) and two independent retinal specialists (HMV and BJL) for description and analysis of important iOCT findings. For statistical analysis to compare categorical data points, Fisher’s exact test was performed using GraphPad Prism 7 (La Jolla, USA). A p-value less than 0.05 was considered statistically significant.

## Results

Twenty-one eyes underwent subretinal gene therapy as part of six different clinical trials during a one-year study period. Of these, 19 eyes were included in the study for analysis. Of the two eyes not enrolled in the study, one was excluded due to failure to create the subretinal bleb using BNT during the surgery and the other eye due technical issues related to iOCT system, making it impossible to collect images. The exact reason for the unsuccessful subretinal bleb creation in one case is unknown. The surgeon multiple attempts but was unable to detach the retina. The mean age was 41.34 years, with a range from 16 to 68 years. There were 11 men (58%) and eight women (42%). Surgical characteristics of the study are summarized in Table 1.

**Table 1:** Surgical characteristics of study participants

Variables	Data
Subretinal pre-bleb procedure	
Performed	13 (68)
Not-performed	6 (32)
Medication injected as read from the injection syringe, vol. (µl)	
Mean (standard deviation)	220.52 (132.80)
Minimum	20
Maximum	450
Subretinal bleb location	
Superior temporal	16 (84)
Nasal	1 (5)
Temporal	2 (11)
Subretinal bleb shape	
Symmetric	4 (21)
Asymmetric	15 (79)
Subretinal bleb with macular detachment	
Present	17 (89)
Not-Present	2 (11)
Needle tip	
Sharp	10 (53)
Blunted	9 (47)
Method of medication delivery	
Automatic	11 (58)
Manual	8 (42)

Data are no. (%) unless otherwise indicated

Intraoperative OCT allowed for cross sectional visualization of the neurosensory retinal detachment and delivery of viral vector into the subretinal space in all 19 cases. Grey-scale display iOCTs were used in six (32%) cases and color scale in 13 (68%). The most important iOCT findings are summarized in Table 2.

**Table 2:** Intraoperative OCT characteristics and findings

Variables	Data
iOCT display	
Grey scale	6 (32)
Color scale	13 (68)
Needle indentation immediately before bleb creation	
No visible indentation	3 (16)
Partial indentation	6 (31)
Full indentation	10 (53)
Directional reflectivity	
Present	9 (47)
Absent	10 (53)
Subretinal air bubble	
Absent	9 (47)
Occluding retinotomy	7 (37)
Not occluding retinotomy	3 (16)
Open retinotomy	
Identified	14 (74)
Not identified	5 (26)
Medication reflux	
Identified	2 (11)
Not identified	17 (89)

*iOCT* intraoperative OCT

Data are no. (%) unless otherwise indicated

**Fig. 1:**
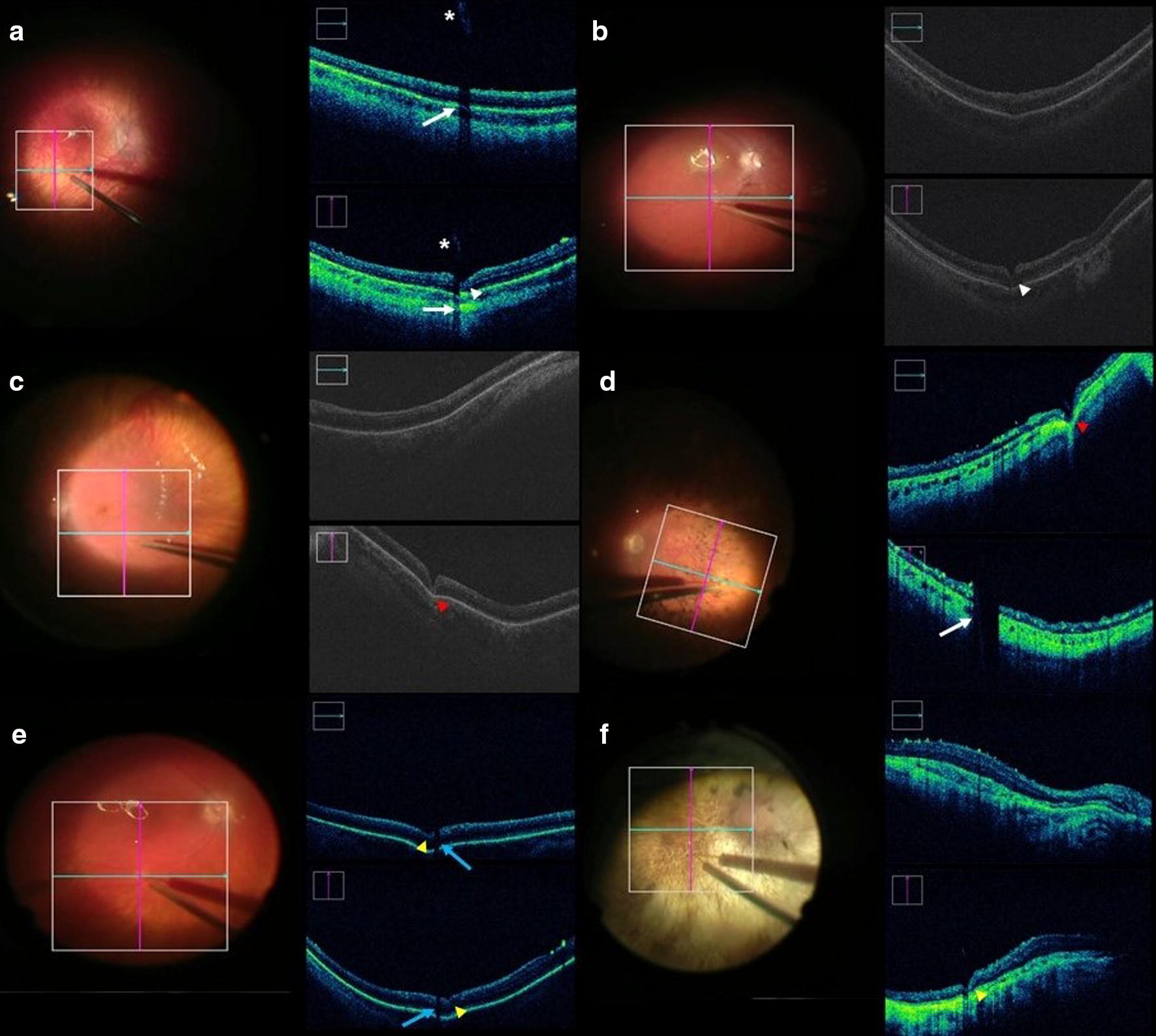
Photographs and intraoperative OCT *(i*OCT) images obtained during gene therapy surgery. **a–f***i*OCT image obtained during initial step of subretinal injection with (**a**, **b**) unsuccessfully attempted to create the bleb with visible bubbles in the vitreous (color photograph) and incomplete retinal indentation (*arrowhead*) with blunted needle tip (*asterisk*) and corresponding shadow of subretinal cannula (*arrow*). **c**, **d** Blunted needle complete indentation/penetration of the retina associated with RPE indentation before successful subretinal injection (*red arrowhead*), and (**d**) corresponding shadow of subretinal cannula (*arrow*). **e**, **f** Sharp needle partial indentation/penetration of the retina associated with RPE partial indentation before successful subretinal injection (*yellow arrowhead*), and corresponding shadow of subretinal cannula (*blue arrow*)

In an effort to better understand factors involved in subretinal bleb formation, we compared two different types of needle tips and their interaction with the retina, during the delivery of the medication (Fig. 1). Of the 19 successful blebs created, only 16 had iOCT images that allowed evaluation of penetration depth. For these 16 eyes, BNT was used in seven (44%) and SNT in nine (56%).

**Fig. 2:**
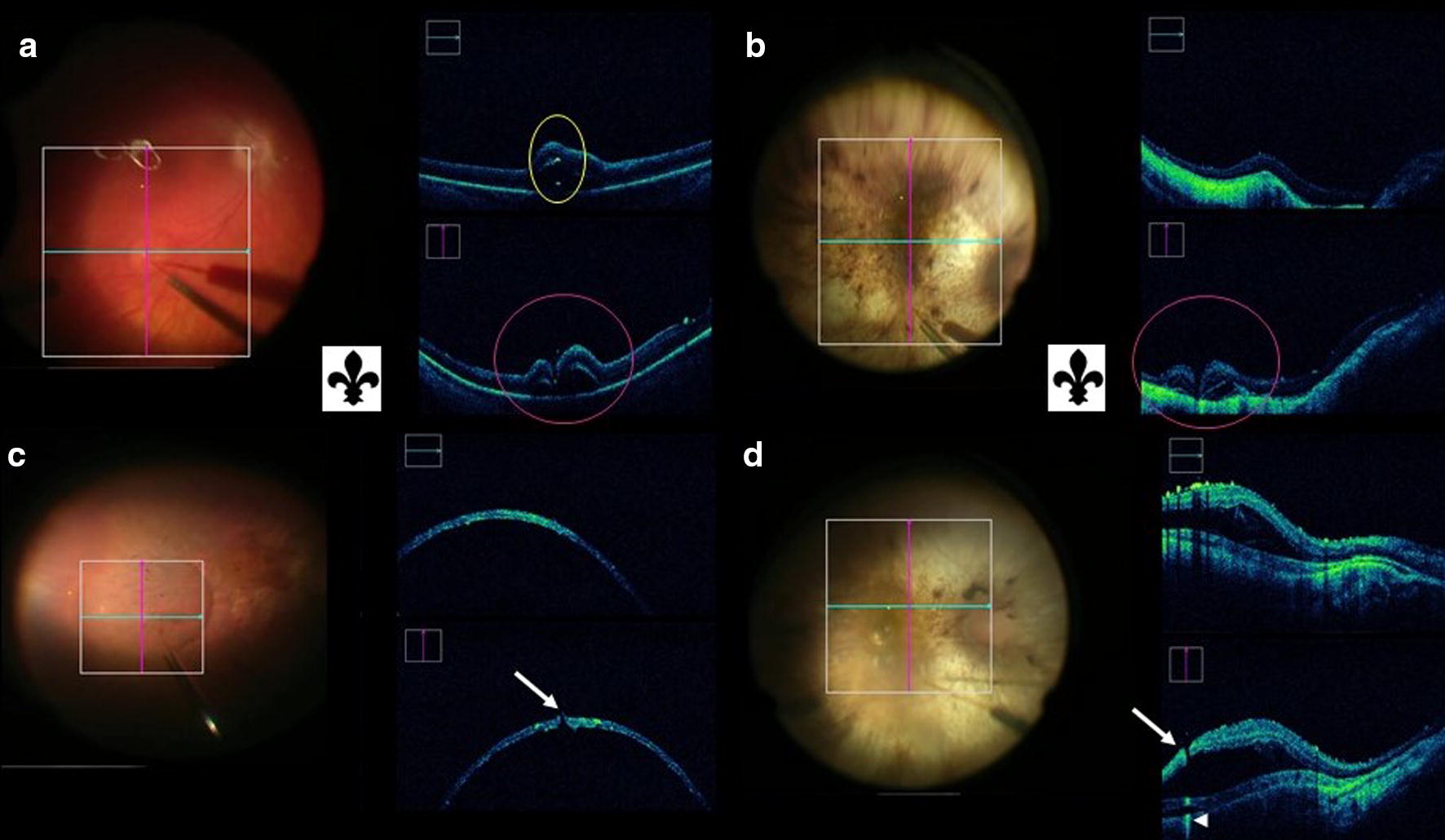
Photographs and intraoperative OCT (*i*OCT) images obtained during gene therapy surgery. **a**, **b** “Fleur-de-lis” sign during initial subretinal injection procedure (*pink circle*) with (**a**) hypereflective image due to air bubble wall reflection—double hypereflective sign *(yellow circle*). **c**, **d***i*OCT image obtained during final step of subretinal injection with visible open retinotomy (*arrow*) and associated transmitted hypereflective image (*arrowhead*)

BNT resulted in an effective bleb creation in all seven eyes when a complete indention/penetration of the neurosensory retina with temporary indentation of the RPE/choroid (compression of the neurosensory retina and RPE/choroid with the needle tip) was performed (Fig. 1d–f). In comparison, SNT required temporary complete indentation only on three eyes (33.3%) and partial indentation was adequate for bleb creation in the other six eyes (66.6%) (Fig. 1g–i). There was a significant association between the use of BNT and the need to temporary completely indent the RPE/choroid to create a subretinal bleb (p = 0.0114).  A distinct sign related with the initiation of subretinal bleb and the interaction of the neurosensorial retina with needle tip was visualized and described as “Fleur-de-lis” sign (Fig. 2a–c).

We used iOCT to examine the incidence of open retinotomies in the final bleb when that was created with a BNT versus a SNT (Fig. 2d–f). Fourteen (74%) out of 19 eyes had an open retinotomy visible in the final bleb, after the medication injected, during the surgical procedure. Of these, ten (71%) followed the use of a SNT and four (29%) followed the use of a BNT. Thus, there was a significant association with the use of SNT and an open retinotomy (p = 0.0108).

**Fig. 3:**
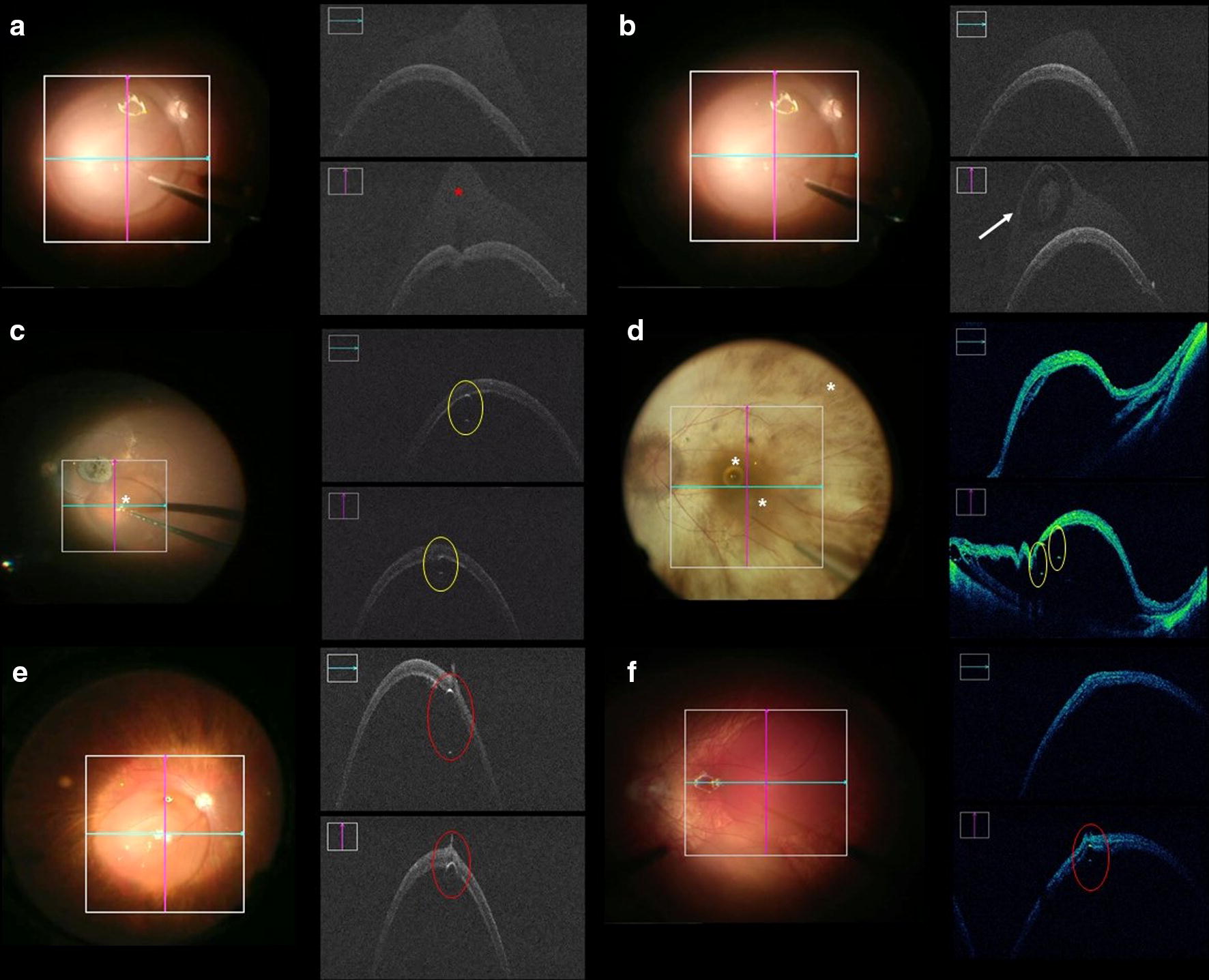
Photographs and intraoperative OCT (*i*OCT) images obtained during gene therapy surgery. **a**, **b***i*OCT image obtained during subretinal injection with (**a**) visible bleb indentation and hypereflective image from medication contrast at the surface of the bleb (medication secondary to a failure attempt to create the bleb) (*red asterisk*) and **b** mixed hyper/hyporeflective image secondary to medication reflux (*arrow*). **c**, **d** Hypereflective image due to air bubble wall reflection - double hypereflective sign (*yellow circle*). **e**, **f** Hypereflective image due to air bubble wall reflection - double hypereflective sign occluding retinotomy (*red circle*)

The observation of medication reflux from the subretinal bleb to the vitreous cavity was an uncommon finding using the iOCT. Only two cases (11%) had visible medication reflux through the retinotomy at the end of the procedure (Fig. 3a, b). No statistically significant correlations between the presence of reflux and the pre-bleb technique, needle tip or the presence of an open retinotomy were identified.

Air bubbles were present in the subretinal space in 10 cases (53%). The reflection from the anterior and posterior air bubble wall resulted in a characteristic double hypereflective sign (Fig. 3c–e). Among the 10 cases with subretinal air bubbles, seven were found to occlude the inner aspect of the retinotomy (Fig. 3f–h). There was no statistically significant relationship between the presence of air bubbles and the absence of visible reflux in our series (p = 0.0867).

**Fig. 4:**
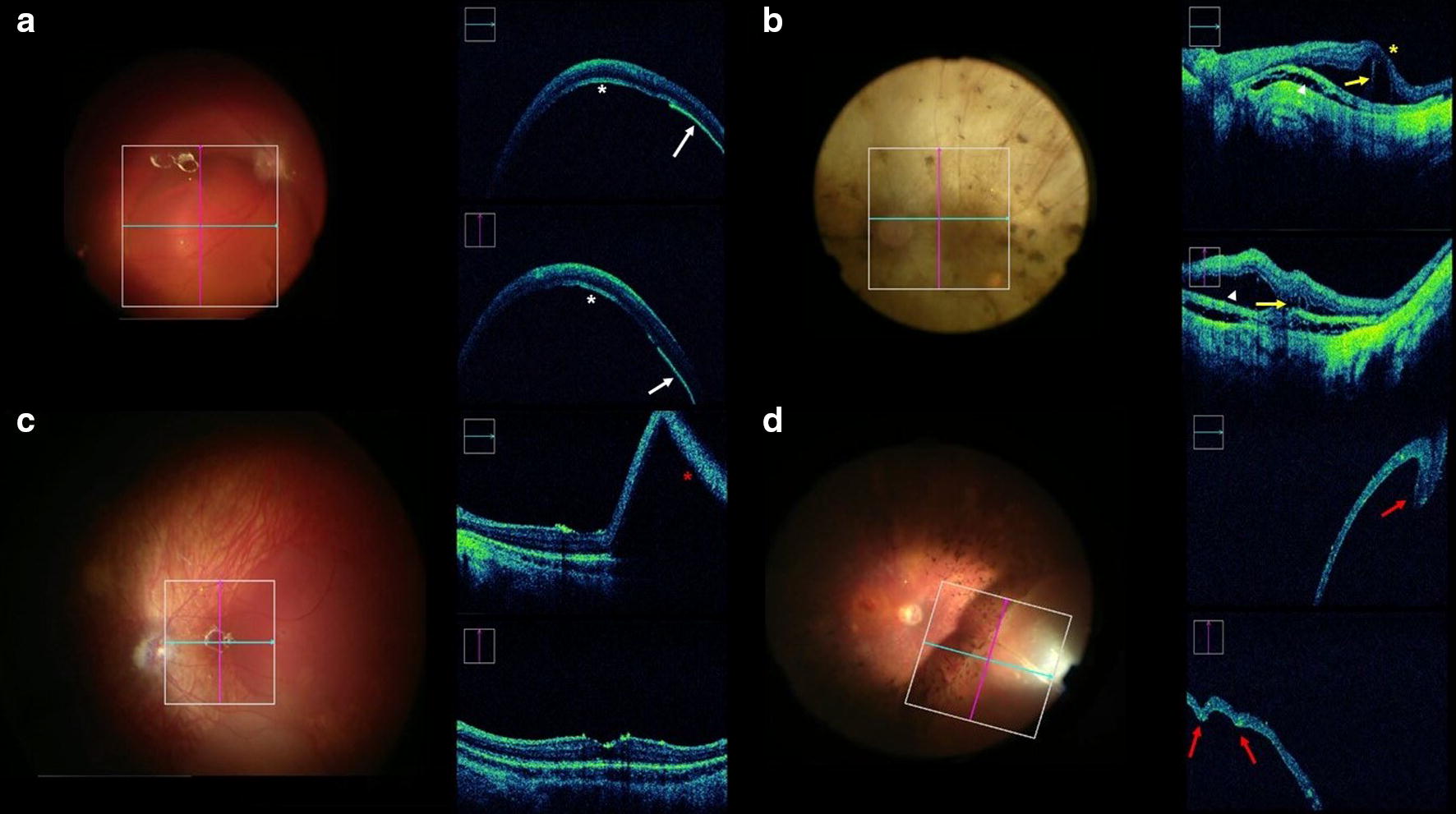
Photographs and intraoperative OCT (*i*OCT) images obtained during gene therapy surgery. **a***i*OCT image obtained after subretinal injection with directional reflectivity image in the posterior surface of the bleb (*asterix*) with associated RPE separation (*arrow*). **b** Intraoperative OCT image obtained after subretinal injection showing a suprachoroidal fluid (*arrowhead*), associated with retinal schisis (*arrowhead and yellow arrow*) and foveal inversion (*yellow asterix*). **c** Mirror artifact with the *i*OCT image focus in the attached retina and in the retinal bleb (*red asterix*). **d** Retinal fold present at the peripheral retina (*red arrow*)

Directional reflectivity was present in nine (47%) patients, always at the outer neurosensory layers of the subretinal bleb (Fig. 4a–c). Retinal pigment epithelial separation (1 case—5%), retinoschisis (7 cases—37%) and suprachoroidal fluid (5 cases—26%) were other isolated findings identified by iOCT (Fig. 4d, e). Mirror artifact occurred in isolated cases, mainly during the subretinal injection and in the transition between attached and detached retina (Fig. 4f). The cannula shadow obscured iOCT visualization of needle tip penetrating the retina in three (16%) cases. Foveal inversion sign was identified in cases with foveal detachment (Fig. 4e) and retinal folds were observed in two (11%) cases (Fig. 4g).

## Discussion

This study represents the largest retrospective series evaluating the features of microscope-integrated iOCT during subretinal gene therapy surgery. Surgeon and skilled assistant directed iOCT scanning to areas of interest and intraoperative recording of surgical events have led to the discovery of novel findings and improved our understanding of retinal bleb creation.

Our results indicate that effective retinal penetration seems to be essential for the success of retinal bleb creation and medication delivery (Fig. 1). Excessively deep penetration of the needle tip with associated blanching can result in hemorrhage, cannula tip obstruction, RPE damage and suprachoroidal delivery of the medication, while too shallow penetration can result in intraretinal hydration and retinoschisis during delivery of the medication. Also, the degree of retinal atrophy in the area of injection can interfere with those complications and be related with more cases of hemorrhage and cannula tip obstruction. Multiple attempts to create subretinal bleb can led to localized neurosensory retinal trauma and enlargement of the retinotomy. All these characteristics are better evaluated with the use of iOCT, reinforcing the importance of this device during the initiation of the subretinal bleb in gene therapy surgeries.

Visualization of the fleur-de-lis sign can confirm initiation of the bleb formation (Fig. 2). We found that when using a BNT, complete temporary indentation of the retina and RPE/choroid was needed for successful medication delivery (Fig. 1). All the successful bleb creation with a BNT was preceded by effective indentation, and ineffective indentation of the RPE was observed during all three failed attempts to creating a subretinal bleb using BNT.

We found that successful subretinal bleb creation using a SNT required only partial retina and RPE/choroid indentation (Fig. 1). Previous discussion about the influence of temporary indentation of the choroid during subretinal gene therapy did not compare the types of needle tips used [23]. The greatest penetrating ability of SNT engender less need for retinal compression but may be associated with a higher risk of retinal hemorrhage, RPE penetration, and choroidal hemorrhage. One case of subretinal hemorrhage was observed in association with SNT whereas no cases were associated with BNT. Fortunately, the hemorrhage was localized, less than one disc diameter in size and without detectable visual or functional consequences.

We found a statistically significant correlation between the use of SNT and the rate open retinotomies (Fig. 2). Ehlers et al [15] described similar disturbances in the retinal architecture that was related to potential areas of previous retinal penetration needle tracks in post-tPA surgery iOCT images, and Gregori et al. [19] showed visible open retinotomy. Despite the use of SNT makes it easier and quicker to penetrate the retina in some cases and the lack of significant correlation between visible open retinotomy and medication reflux in our study, this possibility cannot be discarded.

Visible open retinotomy can be related to other factors different from needle tip characteristics. Surgeon’s hand movement with the needle tip inside the retinotomy, retinal stretching during the medication injection, the total volume of medication delivered in the subretinal space and the rate of retinal atrophy in the area treated can act as confounding factors. The same surgeon performing all surgeries, the use of different needle tips in each type retinal degeneration and the presence of open retinotomies even in cases with low volume injected, reduced the influence of those possible confounding factors in our analysis.

The ability to visualize medication reflux in two cases was another advantage of iOCT (Fig. 3) (see Additional file 1: Video, demonstrating the reflux from the subretinal bleb to the vitreous). The iOCT detection of contrast created by the medication in the vitreous helped to reveal medication reflux in the first case. This hyper-reflective image is similar to that found in the study with triamcinolone and porcine eyes performed by Hsu et al. [17]. The second case shows tissue particles refluxing from the borders of the retinotomy, similar to a case described by Davis et al. [23]. Even with adequate focus and the presence of contrast between medication and avitric cavity, it is conceivable that iOCT is not able to consistently detect medication reflux. A dynamic enlargement of the retinotomy during the medication injection likely represents a reflux condition without medication visualization. The concentration and the type of viral vector used for various treatments could modulate the ability to detect reflux with iOCT.

The commercially available LUMERA 700 with RESCAN device possessed 2 mm capacity in depth analysis what prevented the coverage of the whole area of the bleb intraoperatively, avoiding an ensuing volumetric analysis. This analysis would be important in order to accurately calculate the volume of fluid delivered into subretinal space and it comparison with the volume delivered as read from the injection syringe, thus being important information for estimating of intraoperative medication reflux.

Air bubbles may be identified by iOCT by their double hypereflective sign (Fig. 3). The scattering of light characteristics of an air bubble inside a fluid and the distribution of reflection intensity of the light, can explain the hypereflective signs collected by the iOCT. In addition, there may be distortion of the RPE image and its depth, when the bubble is closer to this location [24]. During the surgical procedure, air bubbles tend to rise to the highest point of the bleb due to buoyancy [25]. However, asymmetry of the subretinal bleb surface can sometimes result in a bubble not localizing at the apex of the bleb, but rather positioning eccentrically to occlude an open retinotomy, possibly preventing reflux of the therapeutic agent into the vitreous. We observed this phenomenon in 70% of cases where air bubbles were present and, despite the easy visualization of the air bubbles without iOCT, it helps in identifying the exact location of the air bubbles regarding relation with retinal layers and position reference to the retinotomy.

The volume of subretinal air bubble could not be calculated, as well as the subretinal medication volume. The air bubbles were generally small and founded in small number. Large amount of air could be harmful to the retina, so visual inspection of the medication in the syringe were carefully performed by the surgeon before all procedures.

Directional reflectivity is an OCT characteristic obtained by changing the orientation of incidence light to the retina, creating contrast of directionally reflective structures [26]. In this study, directional reflectivity between frames of iOCT images were identified in the internal border of the retinal bleb in 9 (47%) eyes (Fig. 4). To our knowledge, this is the first time that this finding has been described in the iOCT. This phenomenon was not due to shifting pupil entry position but occurred because of dynamic changes of the position of the photoreceptors within the retina during the subretinal bleb creation.

Foveal inversion was a dynamic finding associated with foveal detachment during the migration of the medication to the central retina (Fig. 4). During subfoveal medication delivery, careful real-time iOCT examination for foveal inversion was found to be important for safer medication delivery. It is felt that avoiding excessive retinal stretching may prevent complications such as macular holes in patients with very atrophic retina as noted in previous studies [17, 21, 22].

Identification of important dynamic real-time iOCT findings such as intraretinal fluid/schisis, suprachoroidal fluid, RPE separation and retinal folds can be helpful during the procedures (Fig. 4). The RPE separation occurred only in one case in this cohort and distant from the site where the retinotomy were done and the subretinal injection initiated. This separation can be easily confused with directional reflectivity and need to be carefully analyzed to avoid wrong intrasurgical decisions. The dynamic change of the hyper-reflectivity observed at the iOCT directional reflectivity differs it from a real RPE separation. Other example of the importance of dynamic real-time iOCT is it use to differentiate schisis from subretinal fluid. These are cases that exemplify how the iOCT can change the surgeon approach, helping to decide to stop the injection, change the treatment area or just continue the procedure.

Some technical aspect is important in order to acquire good iOCT images. Clarity of the cornea and lens are necessary for optimal images. Improper focus on the surface of the bleb or the attempt to focus on a region of attached retina adjacent to bleb, were responsible for the mirror artifact [27]. The incorrect focus on the superficial retina is responsible for the mirror artifact (Fig. 4). The velocity of expansion of the subretinal bleb and the retinal height during the procedure, makes it difficult the iOCT adequate focus, as showed by Ehlers et al. [15]. This focus problem is also related to limitations regarding the iOCT depth capacity and the difficult dynamic to move the image collector to the right target. One other technical issue was cannula and needle shadow (Fig. 1a) (previously described by Xue et al) [18]. The cannula obscured the needle tip penetrating the retina and indenting the RPE in some cases, and this may make it difficult to determinate the precise time to start the injection of medication.

In using the iOCT system, the surgeon needs time to overcome the challenge of assimilating both the direct axial visualization of the retina through the microscope eyepieces, and the real-time iOCT image display in one microscope ocular. As previously discussed by Ehlers et al. [28] the iOCT image in one microscope ocular can confuse inexperienced surgeons by reducing depth perception and color contrast. The presence of an experienced assistant to operate the iOCT during the procedure helps tremendously by refining the iOCT findings while the surgeon is performing the subretinal injection. Future directions of iOCT imaging may combine automated image tracking and 3-dimensional digital surgical visualization in order to refine gene therapy surgeries [29].

This series is the largest series to date that evaluates iOCT findings during gene therapy surgery. Despite this fact, the present study has some limitations. The small number of participants and the nonrandomized or controlled study population limited further quantitative analyzes and any multivariate comparisons, as well as final conclusions based on the iOCT findings. Due to a learning curve when adopting new technology, obtaining the ideal axial position of the areas of interest was a challenge during the initial surgeries, and may have influenced the frequency of described findings. An additional limitation with the current iOCT display is the inability to readily view both grey and color scales simultaneously. The limited processing speed of the current iOCT was another factor that reduced the capacity to analyze some of the findings such as whether medication reflux was present or not.

The presentation and analyzes of aggregate findings from multiple investigational studies, with different types of retinal degeneration, increased the cohort and made some statistical clarifications possible. The limitation was the impossibility to disclosure the correlation between genotype and intrasurgical. Future studies using bigger cohort, controlled groups, different trained assistant and surgeons is likely to generate additional discoveries and more individualize iOCT findings during gene therapy surgery. Likely future improvements in iOCT technology including faster processing may aid in image interpretation, so that higher quality data can be acquired.

## Conclusion

In conclusion, iOCT was found to be a highly useful tool for surgical maneuvers during retinal gene therapy surgery by providing valuable real-time feedback. Novel intraoperative findings cross-sectional retinal anatomic changes were identified. Future improvements in the iOCT technology such as a better depth capacity, greater area of retina coverage, accurate form to calculate the bleb volume and further imaging studies using iOCT, are likely to help improve and refine our gene therapy delivery techniques and outcomes measurement.

## Supplementary information

**Additional file 1.***i*OCT video, demonstrating the reflux from the subretinal bleb to the vitreous.

## Data Availability

Data is contained within the patient’s medical record and will not be distributed.

## Abbreviations

AKL: Andreas K. Lauer
BJL: Brandon J. Lujan
BNT: Blunted needle tip
HMV: Huber M Vasconcelos Jr
iOCT: Intraoperative optical coherence tomography
IRDs: Inherited retinal dystrophies
OCT: Optical coherence tomography
OHSU-CEI: Oregon Health & Science University-Casey Eye Institute
PSI: Pound-force per square inch
RPE: Retinal pigment epithelial
SD-OCT: Spectral-domain OCT
SNT: Sharp needle tip
tPA: Tissue plasminogen activator
